# Quantification Analysis of 13 Organic Components and 8 Inorganic Elements in *Angelica Sinensis Radix* and Its Different Parts Combined with Chemical Recognition Pattern

**DOI:** 10.1155/2020/8836184

**Published:** 2020-08-31

**Authors:** Xi Li, Yixin Yao, Xiaoxiao Wang, Chang An, Shanshan Gao, Fangtao Xiang, Yangli Dong

**Affiliations:** ^1^Sichuan Institute for Food and Drug Control, Chengdu 611730, China; ^2^Kangmei Pharmaceutical Co., Ltd., Shenzhen 518000, China; ^3^Deyang Food and Drug Safety Inspection and Testing Center, Deyang 618000, China; ^4^School of Pharmacy, Fujian University of Traditional Chinese Medicine, Fuzhou 350122, China; ^5^Affiliated Hospital, Leshan Normal University, Leshan 614004, China

## Abstract

*Angelica Sinensis Radix* (Danggui, DG) is one of the most commonly prescribed traditional Chinese medicines. The organic components include phthalides and phenolic acids. Meanwhile, inorganic elements play an important role in clinical effect. DG and its different parts have different effects. There is no relevant report on the analysis of organic compounds and inorganic elements among them. Therefore, ultra-high-performance liquid chromatography coupled with triple quadrupole mass spectrometry was developed for the simultaneous determination of 13 organic components (8 phthalides and 5 phenolic acids), and 8 inorganic elements were determined by inductively coupled plasma mass spectrometry. The contents of 32 samples were analyzed by orthogonal partial least squares discrimination analysis, hierarchical cluster analysis, and least-significant difference of one-way analysis of variance. The results showed that the differences were significant among DG and its different parts. 11 difference markers (Ca, Z-ligustilide, Mg, Mn, Fe, Na, K, Cu, Zn, coniferyl ferulate, and senkyunolide A) were obtained by variable importance for the project. These difference markers were some different among DG and its different parts, especially Z‐ligustilide, coniferyl ferulate, Mg, Zn, the differences were significant. This study can provide a reference for DG research.

## 1. Introduction

*Angelica Sinensis Radix* (Danggui, DG), the dried root of *Angelica sinensis* (Oliv) Diels. (Umbelliferae), is one of the most commonly prescribed traditional Chinese medicines (TCM). DG is commonly used to enrich blood and regulate menstruation and employed in the treatment of blood deficiency and chlorosis, vertigo and palpitation, irregular menstruation, amenorrhea and dysmenorrhea, asthenia cold abdominalgia, intestinal dryness, and constipation [1]. Modern studies have shown that DG can increase red blood cells and hemoglobin, improve hemorheology and acute myocardial infarction, etc [2–4]. The organic bioactive components mainly consist of phthalides and phenolic acids. Phthalides include senkyunolide I, senkyunolide H, Z-ligustilide, senkyunolide A, etc. Phenolic acids comprise ferulic acid, chlorogenic acid, caffeic acid, and coniferyl ferulate [5–7]. In recent years, numerous studies have found that the efficacy of TCM is not only related to the organic components but also closely related to inorganic elements. In the participation and regulation of metabolism, inorganic elements also represent an important factor for the exertion of pharmacological effects [8, 9]. Mn-superoxide dismutase (Mn-SOD) has a strong correlation with epithelial ovarian tumors [10]. The low ratio of Cu to Zn is prone to hyperlipidemia and coronary heart disease, and Cu deficiency is an important risk factor of coronary heart disease [11, 12]. In ischemic diseases, the excessive increase of [Ca^2+^]_i_ in myocardium will lead to calcium overload and cell death. Ca^2+^ in cardiomyocytes is mainly involved in the excitation contraction coupling of myocardium. Meanwhile, Ca^2+^ homeostasis is regulated by a variety of proteins, including Na^+^/Ca^2+^ exchanger, etc [13, 14]. The content of serum Mg^2+^ and Ca^2+^ is low in patients with cerebral infarction [15, 16]. Fe enriches blood and is related with heart failure [17]. Modern research indicates that the efficacy of DG is strongly related with uterus disease and cardiovascular and cerebrovascular diseases [18]. It indicates that these inorganic elements are also the bioactive components of DG.

There are different parts of DG: head (H), body (B), and tail (T). Whole DG (W) and its different parts have different effects in TCM [19]. Recent research shows that the contents of bioactive components and pharmacological actions are different in DG and its different parts [20–23]. However, there is no study of DG and its different parts based on organic constituents and inorganic elements at the same time.

Ultra-high-performance liquid chromatography coupled with triple quadrupole mass spectrometry (UHPLC–MS/MS) has the characteristics of high accuracy, high sensitivity and rapid analysis and is suitable for the quantitative determination of minor compounds with complex matrix and serious interference. It had been widely used in the analysis research of TCM [5, 6]. Inductively coupled plasma mass spectrometry (ICP-MS) is a rapid development of element analysis technology in recent years, with the advantages of rapid, accurate, and simultaneous determination of multielement and it is widely used in the inorganic elements of TCM [8, 9].

Therefore, the UHPLC-MS/MS method was developed to simultaneously determine 13 organic components (chlorogenic acid, caffeic acid, vanillin, ferulic acid, senkyunolide I, senkyunolide H, coniferyl ferulate, Z-ligustilide, butylphthalide, senkyunolide A, butylidenephthalide, neocnidilide, and levistilide A) in different parts of DG. 8 inorganic elements, including Na, Mg, K, Ca, Mn, Fe, Cu, and Zn, were simultaneously quantified by inductively coupled plasma mass spectrometry (ICP-MS). The results were analyzed by hierarchical cluster analysis (HCA), orthogonal partial least squares discrimination analysis (OPLS-DA), and least-significant difference (LSD) of one-way analysis of variance (ANOVA). It provides useful information for DG research.

## 2. Materials and Methods

### 2.1. Materials and Reagents

Acetonitrile (HPLC grade) was obtained from Fisher Corporation (Waltham, MA, USA). Glacial acetic acid was analytical grade and acquired from Guangzhou Chemical Reagent Factory (Guangzhou, China). Nitric acid 67% was MOS grade and purchased from Tianjin Kemiou Chemical Reagent Co., Ltd. (Tianjin, China). Standards of chlorogenic acid, caffeic acid, vanillin, ferulic acid, senkyunolide I, senkyunolide H, coniferyl ferulate, Z-ligustilide, butylphthalide, senkyunolide A, butylidenephthalide, neocnidilide, and levistilide A (purities ≥ 98% by HPLC) were purchased from Chengdu Pufei De Biotech Co., Ltd. (Chengdu, China). Calibration solutions of 1,000 mg·L^−1^ of Na, Mg, K, Ca, Mn, Fe, Cu, and Zn were all purchased from Agilent Technologies Inc. (USA). Internal standard elements, consisting of 1,000 mg·L^−1^ of ^73^Ge, ^115^In, and ^209^Bi, were obtained from National Institute of Metrology (China). All experimental solutions were prepared with ultrapure water (18.2 MΩ·cm^−1^), which was produced by a purification system (Milli-Q Gradient, Millipore, USA).

### 2.2. Apparatus

BSA224S Precision electronic balance was purchased from Beijing Sartorius Scientific Instrument Co., Ltd. (Beijing, China). KQ-500VDE double frequency digital ultrasonic cleaning instrument was purchased from Kunshan Ultrasonic Instrument Co., Ltd. (Kunshan, China). Chromatographic analysis was performed on a Waters Acquity UHPLC system (Waters, Corp., Milford, MA, USA), consisting of a binary pump solvent management system, an online degasser, and an autosampler. Mass spectrometry detection was performed using a Xevo Triple Quadrupole MS (Waters Corp., Milford, MA, USA) equipped with an electrospray ionization source (ESI). The ESI-MS spectra were acquired by using multiple reaction monitoring (MRM). Inorganic elements analysis was performed on Agilent 7800 ICP-MS system (Agilent Technologies Inc., USA). CEM MARS6 microwave digestion apparatus was purchased from BERGHOF Co., Ltd. (CEM MARS6, Berghof Co., Germany).

### 2.3. Samples Collection

DG samples (8 samples, 2-year-olds) were collected from Minxian, Gansu province, and further were identified as dried radix of *Angelica sinensis* (Oliv.) Diels. by chief pharmacist Liu Maogui, director of quality management department, Kangmei Pharmaceutical Co., Ltd. DG samples were divided into H, B, and T. All samples were deposited in the traditional Chinese Medicine Laboratory of Puning Production Base of Kangmei Pharmaceutical.

### 2.4. Determination of Organic Components by UHPLC-MS/MS

#### 2.4.1. Condition of UHPLC-MS/MS

The column was an Agilent Eclipse Plus C18 column (1.8 *μ*m, 50 × 2.1 mm, Agilent), and the column temperature was kept at 35°C. The flow rate was set at 0.3 mL·min^−1^. The injection volume was 2 *μ*L. 0.1% formic acid (V/V) was selected as mobile phase A, and acetonitrile was selected as mobile phase B. The linear gradient elution of A was performed as follows: 5%A at 0–2 min, 5%–25% A at 2–5 min, 24%–45% A at 5-6 min, 45%–70% A at 6–12 min, and 70%–100% A at 12–15 min.

The ES^+^ mode conditions of MS analysis were set as follows: capillary voltage, 2.0 kV; source temperature, 150°C; desolvation temperature, 500°C; cone gas flow, 20 L/h; and desolvation gas flow, 1000 L/h. The ES^−^ mode conditions were as follows: capillary voltage 2.0 kV; source temperature 72°C; desolvation temperature 350°C; cone gas flow 1 L/h; and desolvation gas flow 650 L/h. The cone voltage and collision energy were set to match the MRM of each compound [24]. The dwell time was automatically set by Mass Lynx software. The summary of MS/MS detection parameters is given in Table 1.

#### 2.4.2. Preparation of Sample

Each dried material was pulverized to 65 mesh. Approximately 0.5 g of pulverized powder was accurately weighted, then extracted with 25 mL methanol by ultrasound extraction (300 W of efficiency, 45 kHz of frequency) for 30 min, cooled to room temperature, and supplemented weightlessness. The extraction solution passed through a filter (0.22 *μ*m mesh size).

### 2.5. Determination of Inorganic Elements by ICP-MS

#### 2.5.1. Sample Pretreatment

All glass instruments and polytetrafluoroethylene (PTFE) digestion tank were soaked about 6 h with 10% (V/V) nitric acid before the experiment. Each dried material was pulverized to 50 mesh. Approximately 0.2 g of pulverized powder was accurately weighted and placed in the PTFE digestion tank, and then 8 mL of nitric acid was added in the fume hood, and the samples were sealed and stayed overnight. The next day, all samples were placed in a microwave digestion apparatus and processed according to the set digestion procedure. The digestion conditions are shown in Table 2. After digestion, the samples were cooled to room temperature, the digestion tank was removed, the acid was taken out of the fume hood, and deionized water was transferred to a constant volume of 50 mL. Simultaneously, 8 mL concentrated nitric acid were used as blank. The supernatant was collected and passed through a filter (0.22 *μ*m mesh size).

### 2.6. Solutions Preparation

The 21 reference compounds were respectively prepared by completely dissolving in methanol, and their concentrations were as follows: chlorogenic acid, 0.0217 mg/mL; caffeic acid, 0.0077 mg/mL; vanillin, 0.0156 mg/mL; ferulic acid, 0.0678 mg/mL; senkyunolide I, 0.0226 mg/mL; senkyunolide H, 0.0075 mg/mL; senkyunolide A, 0.0846 mg/mL; coniferyl ferulate, 0.1744 mg/mL; Z-ligustilide, 0.0954 mg/mL; butylidenephthalide, 0.0142 mg/mL; neocnidilide, 0.0316 mg/mL; levistilide A, 0.0159 mg/mL. Na, Mg, K, Ca, Mn, Fe, Cu, and Zn were 100 *μ*g/mL. All the stock solutions were stored at 4°C before analysis.

### 2.7. Method Validation and Sample Determination

UHPLC-MS/MS method: This stock solution was further diluted to a series of different concentration solutions with methanol for the establishment of the calibration curves. These mixture standard solutions were injected in triplicate, and calibration curves were constructed by plotting the peak area (*Y*-axis) versus the concentration (*X*-axis) of each analyte.

#### 2.7.1. ICP-MS Method

The mixed standard mother liquor of Na, Mg, K, Ca, Mn, Fe, Cu, and Zn was taken and diluted to 5, 10, 20, 50, and 100 *μ*g/mL with 10% HNO_3_. Meanwhile, 10% HNO_3_ was used as blank. A standard solution was prepared according to the level of the measured elements in the sample. A series of mass concentration standard solutions of eight inorganic elements were determined. ^73^Ge, ^115^In, and ^209^Bi internal standard solutions were added, and standard blank solution was prepared at the same time. With the standard mass concentration as abscissa (*X*) and the ratio of peak signal value to reference peak response value of internal standard elements as longitudinal coordinate (*Y*), the standard curve was drawn, and the regression equation, correlation coefficient, and linear range of each element standard were obtained.

The limit of detection (LOD) and limit of quantitation (LOQ) were determined by a series of diluted standard solutions until the signal-to-noise (S/N) ratio was approximately 3 and 10, respectively. The precision of the method was determined by the analysis of six consecutive injections using the same sample solution. Repeatability of the method was evaluated by analyzing six samples from the same source using the developed method. The stability was evaluated by storing the sample solutions at 25°C, then analyzed at 0, 2, 4, 6, 8, and 12 h, respectively. To evaluate accuracy, a recovery test was conducted by standard protocol and calculated by the formula [(total detected amount − original amount)/spiked amount] × 100%. Variations are expressed in terms of the relative standard deviation (RSD) of the measurement in all tests.

The quantitative determination of 13 organic constituents of DG and its different parts was performed under the optimal condition by UHPLC-MS/MS and that of 8 inorganic elements was performed by ICP-MS. The results of samples were shown as mean (mg/g) ± SD (%).

### 2.8. Statistical Analysis

The result of analysis was performed using OPLS-DA, HCA, and LSD of one-way ANOVA. OPLS-DA and HCA were carried out by SIMCA-P 14.0 software (Umetrics AB, Umea, Sweden). The sample variation could be assessed by OPLS-DA, the parameters of the modeling (*R*^2^ and *Q*^2^ values) explain the quality of the fitting model. In HCA, a dendrogram was obtained to characterize the classification result of the samples by Ward's linkage as cluster method. LSD of one-way ANOVA was carried out by SPSS 19.0 software (Palo Alto, CA, USA) and the differences were considered statistically significant when *P* < 0.05 and were considered extremely significant when *P* < 0.01.

## 3. Results and Discussion

### 3.1. Optimization of MS Conditions

**Figure 1:**
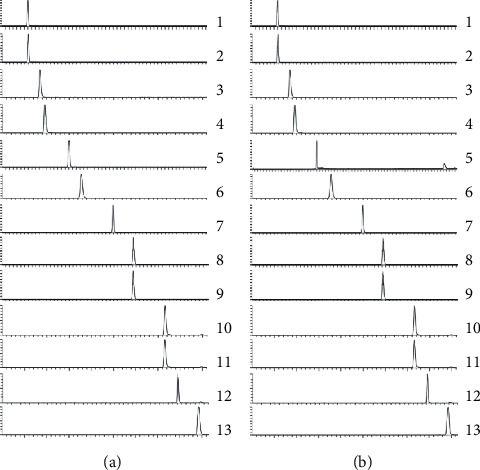
MRM chromatogram of 13 compounds investigated in the mix standards (a) and sample (b) of DG. (1) Chlorogenic acid, (2) caffeic acid, (3) vanillin, (4) ferulic acid, (5) senkyunolide I, (6) senkyunolide H, (7) coniferyl ferulate, (8) senkyunolide A, (9) butylphthalide, (10) butylidenephthalide, (11) Z-ligustilide, (12) neocnidilide, and (13) levistilide A.

In order to obtain an accurate and sensitive quantitative method by UHPLC-MS/MS, individual solutions of all standard compounds were determined with the electrospray ionization (ESI) source by a full-scan mass spectrometry (MS) method and in both positive and negative modes to optimize the parameters of cone voltage (CV) and collision energy (CE) with the highest sensitivity. Meanwhile, multiple reaction monitoring (MRM) from MS/MS spectrum was chosen when the most abundant, specific, and stable fragment ions appeared. The detailed information of retention time (*t*_R_), MS information, CV, and CE for each analyte was listed in Table 1 and Figure 1.

### 3.2. Optimization of ICP-MS Conditions

The working parameters of ICP-MS were set by automatic tuning, and the working parameters of the instrument were optimized based on sensitivity, background, stability, and other indicators. The measurement conditions are shown in Table 3. The measuring method used was the standard curve method, and the reading method was peak strength. Using ^73^Ge, ^115^In, and ^209^Bi as internal standards to monitor the change of the signal can effectively overcome the drift of the instrument signal and correct any matrix effects.

### 3.3. Validation of Methodology

#### 3.3.1. UHPLC-MS/MS Method

Calibration curves were developed from the chromatographic peak area relative to the weights of each compound, respectively. Also, limit of detection (LOD, S/N = 3) and limit of quantification (LOQ, S/N = 10) were calculated. The results showed that the *R*^2^ value of the calibration curves of all components were above 0.9997. The precision, repeatability, stability (12 h), and average recovery (low, medium, high) were evaluated by the contents of 13 constituents, with six samples in parallel, and they were expressed as RSD (%) within 5%. Because coniferyl ferulate, Z-ligustilide, butylphthalide, senkyunolide A, butylidenephthalide, neocnidilide, and levistilide A were unable, the interday results of precision were beyond 5%. The result is shown in Table 4.

#### 3.3.2. ICP-MS Method

The linearity of each element was good (*R*^2^ were above 0.9992) and within the range of 0–100 *μ*g/mL. Also, limit of detection (LOD, S/N = 3) and limit of quantification (LOQ, S/N = 10) were calculated. The accuracy, repeatability, stability (24 h), and recovery were evaluated based on the contents of eight inorganic elements, with six samples in parallel, and were expressed as RSD (%) within 5%. The results are shown in Table 4.

### 3.4. HCA

**Figure 2:**
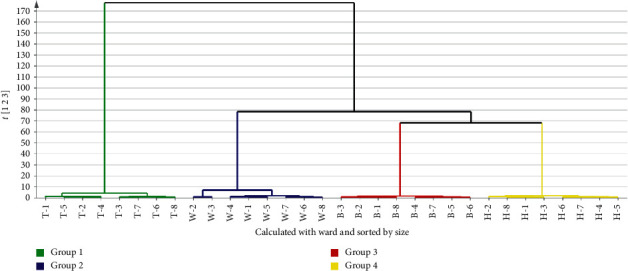
The result of dendrogram by HCA (group 1, T-1∼T-8; group 2, W-1∼W-8; group 3, H-1∼H-8; group 4, B-1∼B-8).

To compare the difference among DG and its different parts, HCA was performed. A total of 32 samples were selected for analysis, while the contents of 21 compounds (Table 5) were selected as variables. In the dendrogram of HCA (Figure 2), all samples were mainly divided into four categories. Firstly, T and others were respectively belonged to one category, indicating that the difference was significant between T and others, respectively. Secondly, W were clustered into one category, B and H were clustered into other category, indicating that the difference was some significant between W and B, H, respectively. Then, B and H were clustered into category, respectively, indicating that the difference was significant between B and H. It indicated that the differences were significant among DG and its different parts.

### 3.5. OPLS-DA

**Figure 3:**
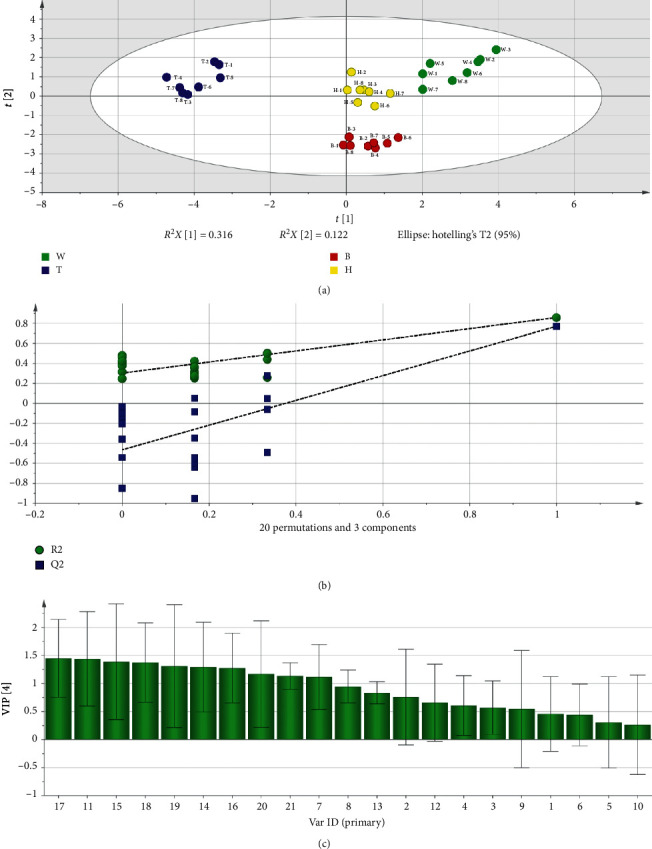
The results of statistical analysis by OPLS-DA: (a) score scatter plot; (b) permutation; (c) VIP plot.

To further compare the difference among DG and its different parts, OPLS-DA was performed. A total of 32 samples were selected for analysis, while the contents of 21 compounds (Table 5) were selected as variables. In the OPLS-DA, the first three principal components were selected, *R*^2^*X* (cum) was 0.612, *R*^2^*Y* (cum) was 0.922, and *Q*^2^ (cum) was 0.85, and we generated score scatter plot, permutation, and variable importance plot (Figure 3). In the score scatter plot (Figure 3(a)), all samples were divided into four parts, indicating that the differences were significant among the different parts of DG and DG. In the permutation (Figure 3(b)), *R*^2^ was 0.303, *Q*^2^ was −0.465, and the values of left are lower than the right, indicating that the model was accurate and predictive. In the variable importance plot (VIP) (Figure 3(c)), the value of VIP in decreasing order was as follows: Mg (1.32) = Ca (1.32) > Z-ligustilide (1.30) > Na (1.28) > Mn (1.26) > Fe (1.25) = K (1.25) > Zn (1.19) > Cu (1.14) > coniferyl ferulate (1.13) > senkyunolide A (1.06) > levistilide A (0.94) > caffeic acid (0.88) > neocnidilide (0.73) > Vanillin (0.69) > ferulic acid (0.64) > senkyunolide H (0.60) > butylphthalide (0.53) > senkyunolide I (0.52) > chlorogenic acid (0.49) > butylidenephthalide (0.41). When VIP > 1, 11 constituents (Ca, Z-ligustilide, Mg, Mn, Fe, Na, K, Cu, Zn, coniferyl ferulate, Senkyunolide A) were acquired, indicating that these constituents were difference markers among DG and its different parts.

### 3.6. Analysis of 11 Difference Markers among DG and Its Different Parts

**Figure 4:**
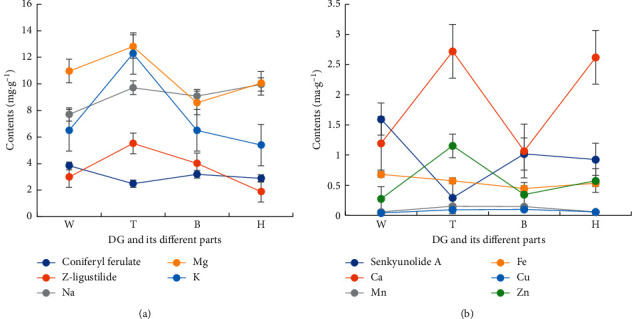
The comparison of difference markers among DG and different parts.

Z-ligustilide, T > B > W > H; coniferyl ferulate and senkyunolide A, W > B > H > T; Mg, T > W > H > B; Zn, T > H > B > W; Ca, T > H > W > B; Mn, T > B > H > W; Fe, W > T > H > B; Na, H > T > B > W; K, T > B > W > H; Cu, B > T > H > W (Figure 4). A total of 32 samples were selected for analysis, while the contents of 11 QDMs were selected as variables and LSD of one-way ANOVA was performed (Table 6). It indicated that there were some differences in the 11 difference markers among DG and its different parts, especially Z-ligustilide, coniferyl ferulate, Mg, and Zn; the differences were significant.

TCM is usually prepared by boiling herbs in water, and it is impossible to obtain all the chemical components of herbs. The emerging potential methods of pretreatment and extraction of active components with ammonia and hydrogen peroxide [25–27] and presoaking [28–30] may bring unexpected insights into the material basis of TCM. For DG, Z-ligustilide, coniferyl ferulate, and senkyunolide A, they were liposoluble components, their solubility was low in decoction, especially coniferyl ferulate; it was easily hydrolyzed to ferulic acid [31]. In addition, TCM contains water, and the determination of water content may be performed before extraction [32], which contributes to accurate determination of active components in future research.

## 4. Conclusions

The UHPLC-MS/MS method and ICP-MS method are accurate and reliable methods for the quantification of 21 bioactive components (13 organic components and 8 inorganic elements) in DG and its different parts. The differences were significant among DG and its different parts. The difference markers were 11 bioactive constituents (Ca, Z-ligustilide, Mg, Mn, Fe, Na, K, Cu, Zn, coniferyl ferulate, and senkyunolide A). This study can provide a reference for DG research.

## Figures and Tables

**Table 1:** UHPLC-MS/MS parameters for MRM of compounds of sample.

No.	Compound	Molecular formula	*t*_*R*_ (min)	[M + H]^+^(m/z)	[M − H]^−^(m/z)	MS/MS fragments ions	Cone voltage (V)	Collision energy (eV)
1	Chlorogenic acid	C_16_H_18_O_9_	1.89	—	353	191	31	30
2	Caffeic acid	C_9_H_8_O_4_	1.91	—	179	135, 134	32	34
3	Vanillin	C_8_H_8_O_3_	2.56	153	—	105, 93	31	28
4	Ferulic acid	C_10_H_10_O_4_	2.71	195	—	117, 89	32	26
5	Senkyunolide I	C_12_H_16_O_4_	4.64	225	—	189, 119	23	22
6	Senkyunolide H	C_12_H_16_O_4_	4.92	225	—	189, 119	23	21
7	Coniferyl ferulate	C_20_H_20_O_6_	6.98	—	355	163, 134	23	26
8	Senkyunolide A	C_12_H_16_O_2_	7.39	193	—	91	21	23
9	Butylphthalide	C_12_H_14_O_2_	7.41	191	—	145	29	14
10	Z-ligustilide	C_12_H_14_O_2_	9.68	191	—	115, 91	23	31
11	butylidenephthalide	C_12_H_12_O_2_	9.70	189	—	89	19	21
12	neocnidilide	C_12_H_18_O_2_	10.57	195	—	91	27	33
13	Levistilide A	C_24_H_28_O_4_	11.87	357	—	191	30	23

**Table 2:** Microwave operating conditions for the digestion of samples.

Time (min)	Temperature (℃)	Power (W)
0	Ordinary temperature	0
10	130	1550
15	130	1550
25	165	1550
35	165	1550
40	180	1550

**Table 3:** Optimum ICP-MS operating conditions for the analysis of samples.

Instrument parameter	Condition
Plasma radio frequency power	1550 W
Plasma gas	15 L/min
Auxiliary gas flow rate	1 L/min
Spray flow rate	1 L/min
Compensation/dilution gas	1 L/min
Spray chamber temperature	2°C
Peristaltic pump speed	0.3 rps
Integral time	1 s
delay time	1 s
Repetition times	3
Isotopes measured	Na, Mg, K, Ca, Mn, Fe, Cu, Zn
internal standards	^73^Ge, ^115^In, ^209^Bi

**Table 4:** Analytical parameters of the quantitation method.

No.	Compound	Linear	*R* ^2^	Range (*μ*g/ml)	LOD (ng/ml)	LQD (ng/ml)	Precision (RSD, %)		Stability (RSD, %)	Reproducibility (RSD, %)	Recovery					
							Intra-day	Inter-day			Low		Medium		High	
											Average (%)	RSD (%)	Average (%)	RSD (%)	Average (%)	RSD (%)
1	Chlorogenic acid	*Y* = 6001.7*X* + 0.0229	0.9999	0.339–21.7	1.90	5.76	0.32	0.10	0.66	0.71	97.32	1.12	98.60	1.12	98.33	1.02
2	Caffeic acid	*Y* = 23817*X* − 0.008	0.9999	0.12–7.7	0.71	2.15	0.25	0.21	0.39	0.45	99.28	1.09	99.28	0.75	101.23	0.56
3	Vanillin	*Y* = 24882*X* − 1.2387	1.000	0.244–15.6	4.23	12.81	0.71	0.19	0.58	0.81	102.64	0.98	100.1	1.08	100.76	0.33
4	Ferulic acid	*Y* = 5998*X* − 2.345	0.9998	0.53–67.8	3.2	9.68	0.48	0.22	0.42	0.77	98.29	1.36	101.21	0.89	101.28	0.49
5	Senkyunolide I	*Y* = 31125*X* + 0.9982	1.000	0.177–22.6	4.27	12.58	0.49	0.35	0.61	0.79	100.02	1.02	99.84	1.54	100.99	0.77
6	Senkyunolide H	*Y* = 30991*X* + 1.0871	0.9999	0.116–7.5	2.17	6.63	0.51	0.87	0.82	1.09	101.25	0.98	102.78	1.42	99.87	0.82
7	Coniferyl ferulate	*Y* = 5109*X* + 2.9871	0.9997	2.725–174.4	2.32	6.98	0.32	10.32	1.98	1.77	97.33	2.39	101.55	2.99	96.25	3.98
8	Senkyunolide A	*Y* = 4768.1*X* + 1.216	0.9999	0.661–84.6	0.43	12.3	0.88	6.87	1.67	1.87	98.32	2.01	98.86	2.08	102.34	2.11
9	Butylphthalide	*Y* = 13452*X* + 0.3876	0.9998	0.174–11.2	1.07	3.22	0.58	7.21	0.89	1.26	98.29	1.87	99.22	1.76	98.76	2.88
10	Butylidenephthalide	*Y* = 15552*x* + 0.987	0.9998	0.322–41.2	1.06	3.22	1.21	9.34	0.98	1.88	97.49	2.34	97.33	1.08	99.23	3.72
11	Z-ligustilide	*Y* = 10008*X* − 1.8766	0.9998	0.745–95.4	1.40	5.14	0.88	10.38	2.36	1.87	96.11	1.59	96.07	3.09	96.11	3.77
12	Neocnidilide	*Y* = 16987*x* − 0.0087	0.9998	0.247–31.6	1.56	4.72	0.59	9.28	0.77	1.12	98.34	2.34	98.28	1.49	98.76	2.34
13	Levistilide A	*Y* = 15992*X* + 0.4871	0.9998	0.248–15.9	0.71	2.12	0.76	8.77	0.99	1.65	95.82	1.89	99.65	1.09	99.12	2.97
14	Na	*Y* = 1.3332*X* + 0.1325	0.9996	0∼100	12.3	38.1	0.98	1.30	2.33	1.46	98.45	1.02	97.32	3.01	101.22	1.33
15	Mg	*Y* = 0.0607*X* + 0.0010	0.9992	0∼100	6.60	20.1	1.2	0.99	2.1	2.08	99.35	1.13	102.08	1.63	100.49	1.09
16	K	*Y* = 0.0046*X* + 2.2888*e* − 4	0.9999	0∼100	24.1	74.7	0.45	0.76	1.58	1.47	102.43	2.32	98.23	1.99	102.33	1.29
17	Ca	*Y* = 2.0325*X* + 0.0154	0.9998	0∼100	0.90	2.70	0.98	0.38	0.88	1.88	101.21	1.79	102.89	2.71	99.45	0.98
18	Mn	*Y* = 4.5207*X* + 0.0802	1.000	0∼100	14.2	42.7	1.26	1.33	1.22	2.05	100.92	1.22	96.33	1.68	97.35	1.76
19	Fe	*Y* = 14.5814*X* + 0.0172	0.9996	0∼100	0.40	1.20	0.94	0.96	1.76	2.31	101.24	0.98	98.25	2.38	98.78	1.32
20	Cu	*Y* = 10.7285*X* + 0.0369	0.9999	0∼100	0.60	1.80	0.57	0.77	1.39	1.87	99.73	1.01	101.29	3.09	101.22	0.87
21	Zn	*Y* = 0.8232*X* + 0.0520	0.9998	0∼100	4.30	13.1	1.02	1.03	2.48	1.59	97.65	1.04	103.19	3.18	100.21	0.79

**Table 5:** The contents of 21 effective components (mg/g) of DG and its different parts (mean ± SD).

Sample	1	2	3	4	5	6	7	8	9	10	11	12	13	14	15	16	17	18	19	20	21
W-1	0.11 ± 0.14	0.02 ± 0.03	0.01 ± 0.02	0.10 ± 0.07	0.09 ± 0.06	0.02 ± 0.03	3.73 ± 0.98	1.61 ± 0.22	0.03 ± 0.02	0.34 ± 0.13	3.28 ± 0.56	0.12 ± 0.07	0.06 ± 0.09	8.05 ± 1.23	10.02 ± 0.98	7.29 ± 0.78	1.52 ± 0.88	0.07 ± 0.01	0.76 ± 0.08	0.10 ± 0.08	0.35 ± 0.25
W-2	0.08 ± 0.07	0.02 ± 0.02	0.01 ± 0.01	0.13 ± 0.08	0.11 ± 0.08	0.01 ± 0.02	3.55 ± 1.76	2.02 ± 0.28	0.03 ± 0.01	0.23 ± 0.03	2.92 ± 0.64	0.34 ± 0.23	0.07 ± 0.03	7.05 ± 0.91	11.08 ± 1.32	5.96 ± 0.88	0.93 ± 0.91	0.06 ± 0.09	0.70 ± 0.17	0.05 ± 0.02	0.23 ± 0.12
W-3	0.12 ± 0.22	0.01 ± 0.01	0.02 ± 0.01	0.14 ± 0.09	0.08 ± 0.07	0.01 ± 0.01	4.13 ± 1.21	2.21 ± 0.09	0.02 ± 0.01	0.07 ± 0.11	2.78 ± 0.04	0.13 ± 0.08	0.07 ± 0.06	6.23 ± 0.49	11.87 ± 1.43	6.40 ± 1.02	0.94 ± 1.21	0.06 ± 0.01	0.71 ± 0.21	0.03 ± 0.01	0.37 ± 0.15
W-4	0.07 ± 0.09	0.02 ± 0.03	0.02 ± 0.03	0.07 ± 0.08	0.09 ± 0.11	0.02 ± 0.01	3.97 ± 1.09	1.33 ± 0.78	0.02 ± 0.01	0.13 ± 0.09	3.01 ± 0.99	0.22 ± 0.14	0.08 ± 0.04	7.17 ± 1.09	11.61 ± 2.01	6.76 ± 0.69	1.28 ± 1.09	0.05 ± 0.03	0.76 ± 0.37	0.03 ± 0.02	0.27 ± 0.08
W-5	0.10 ± 0.07	0.02 ± 0.03	0.01 ± 0.02	0.10 ± 0.07	0.11 ± 0.81	0.02 ± 0.01	4.04 ± 0.89	0.92 ± 0.12	0.03 ± 0.02	0.24 ± 0.02	3.32 ± 1.01	0.30 ± 0.08	0.06 ± 0.02	8.12 ± 0.81	11.78 ± 2.83	7.40 ± 1.32	0.95 ± 0.34	0.04 ± 0.01	0.66 ± 0.18	0.05 ± 0.03	0.25 ± 0.02
W-6	0.11 ± 0.08	0.01 ± 0.02	0.01 ± 0.01	0.09 ± 0.13	0.10 ± 1.22	0.03 ± 0.01	3.69 ± 0.89	1.47 ± 0.91	0.03 ± 0.02	0.14 ± 0.07	2.21 ± 0.23	0.12 ± 0.09	0.08 ± 0.03	8.95 ± 0.75	10.31 ± 1.94	6.43 ± 0.81	1.14 ± 0.21	0.05 ± 0.08	0.68 ± 0.32	0.02 ± 0.03	0.29 ± 0.09
W-7	0.11 ± 0.13	0.02 ± 0.01	0.02 ± 0.03	0.12 ± 0.08	0.09 ± 0.87	0.01 ± 0.01	3.74 ± 0.43	1.63 ± 0.76	0.01 ± 0.01	0.16 ± 0.08	3.33 ± 0.21	0.02 ± 0.01	0.04 ± 0.01	8.18 ± 1.02	9.96 ± 3.02	6.74 ± 1.31	1.39 ± 0.29	0.07 ± 0.02	0.61 ± 0.34	0.06 ± 0.02	0.22 ± 0.13
W-8	0.09 ± 0.06	0.02 ± 0.03	0.02 ± 0.01	0.10 ± 0.06	0.09 ± 0.98	0.02 ± 0.02	3.82 ± 0.89	1.57 ± 0.68	0.03 ± 0.01	0.10 ± 0.09	3.08 ± 0.82	0.10 ± 0.09	0.06 ± 0.02	7.93 ± 1.13	11.08 ± 0.98	5.05 ± 0.99	1.44 ± 0.72	0.06 ± 0.01	0.56 ± 0.18	0.01 ± 0.02	0.25 ± 0.03

T-1	0.11 ± 0.09	0.03 ± 0.02	0.01 ± 0.02	0.11 ± 0.13	0.11 ± 0.07	0.02 ± 0.03	2.76 ± 0.81	0.78 ± 0.21	0.02 ± 0.03	0.16 ± 0.12	5.84 ± 0.31	0.09 ± 0.04	0.04 ± 0.02	9.12 ± 0.92	14.02 ± 2.77	12.81 ± 0.48	2.78 ± 0.62	0.15 ± 0.09	0.69 ± 0.17	0.11 ± 0.03	1.15 ± 0.31
T-2	0.10 ± 0.13	0.01 ± 0.03	0.01 ± 0.01	0.09 ± 0.14	0.08 ± 0.05	0.01 ± 0.01	2.35 ± 0.32	0.01 ± 0.01	0.02 ± 0.01	0.17 ± 0.09	4.98 ± 0.49	0.03 ± 0.01	0.05 ± 0.07	9.61 ± 0.33	14.30 ± 1.73	12.70 ± 0.89	2.70 ± 0.33	0.17 ± 0.03	0.63 ± 0.12	0.08 ± 0.02	1.18 ± 0.29
T-3	0.08 ± 0.22	0.03 ± 0.02	0.02 ± 0.03	0.09 ± 0.07	0.07 ± 0.06	0.02 ± 0.01	2.23 ± 0.49	0.05 ± 0.03	0.03 ± 0.02	0.10 ± 0.02	5.12 ± 0.39	0.10 ± 0.02	0.02 ± 0.01	9.88 ± 1.32	13.12 ± 1.09	12.09 ± 0.78	3.02 ± 0.81	0.13 ± 0.08	0.39 ± 0.18	0.07 ± 0.04	1.12 ± 0.32
T-4	0.11 ± 0.12	0.03 ± 0.01	0.02 ± 0.01	0.08 ± 0.07	0.10 ± 0.12	0.01 ± 0.01	2.25 ± 0.22	0.14 ± 0.71	0.02 ± 0.01	0.23 ± 0.09	5.23 ± 0.89	0.25 ± 0.12	0.02 ± 0.01	10.05 ± 0.81	12.91 ± 2.31	12.26 ± 1.21	2.48 ± 0.82	0.21 ± 0.01	0.71 ± 0.09	0.09 ± 0.03	1.14 ± 0.45
T-5	0.12 ± 0.14	0.02 ± 0.03	0.03 ± 0.07	0.08 ± 0.12	0.09 ± 0.07	0.01 ± 0.02	2.73 ± 0.11	0.58 ± 0.09	0.04 ± 0.02	0.15 ± 0.07	5.77 ± 1.05	0.19 ± 0.07	0.04 ± 0.02	9.32 ± 1.03	10.96 ± 1.97	11.40 ± 1.38	2.52 ± 1.03	0.16 ± 0.03	0.66 ± 0.22	0.09 ± 0.02	1.11 ± 0.23
T-6	0.14 ± 0.09	0.02 ± 0.01	0.03 ± 0.02	0.08 ± 0.05	0.10 ± 0.11	0.02 ± 0.01	2.83 ± 0.78	0.47 ± 0.31	0.02 ± 0.01	0.11 ± 0.09	5.91 ± 0.89	0.20 ± 0.01	0.04 ± 0.01	9.52 ± 0.81	12.07 ± 1.84	12.30 ± 1.04	2.72 ± 1.09	0.15 ± 0.09	0.61 ± 0.33	0.11 ± 0.01	1.17 ± 0.44
T-7	0.11 ± 0.05	0.03 ± 0.05	0.02 ± 0.01	0.10 ± 0.07	0.12 ± 0.13	0.02 ± 0.02	2.23 ± 0.79	0.24 ± 0.11	0.03 ± 0.01	0.25 ± 0.12	5.49 ± 0.32	0.24 ± 0.11	0.03 ± 0.01	10.06 ± 1.04	12.41 ± 1.93	12.87 ± 0.72	2.70 ± 0.91	0.12 ± 0.09	0.43 ± 0.21	0.10 ± 0.03	1.20 ± 0.32
T-8	0.11 ± 0.04	0.03 ± 0.01	0.02 ± 0.02	0.11 ± 0.03	0.11 ± 0.09	0.02 ± 0.01	2.48 ± 0.56	0.08 ± 0.06	0.03 ± 0.02	0.14 ± 0.17	5.88 ± 1.12	0.18 ± 0.08	0.04 ± 0.02	10.18 ± 0.73	12.72 ± 1.72	11.89 ± 0.43	2.83 ± 0.88	0.15 ± 0.03	0.48 ± 0.12	0.11 ± 0.02	1.15 ± 0.21

B-1	0.11 ± 0.13	0.03 ± 0.05	0.03 ± 0.01	0.10 ± 0.09	0.10 ± 0.02	0.02 ± 0.01	3.33 ± 1.78	0.94 ± 0.02	0.02 ± 0.01	0.09 ± 0.03	4.24 ± 1.29	0.23 ± 0.12	0.07 ± 0.01	9.62 ± 0.83	8.97 ± 0.87	6.75 ± 1.09	1.44 ± 1.42	0.16 ± 0.01	0.46 ± 0.32	0.10 ± 0.03	0.37 ± 0.19
B-2	0.08 ± 0.09	0.03 ± 0.04	0.02 ± 0.01	0.07 ± 0.05	0.09 ± 0.04	0.02 ± 0.02	3.14 ± 1.09	0.89 ± 0.79	0.03 ± 0.02	0.15 ± 0.02	4.19 ± 0.79	0.05 ± 0.02	0.06 ± 0.03	8.96 ± 0.52	7.22 ± 1.22	6.63 ± 1.21	1.28 ± 1.32	0.12 ± 0.03	0.48 ± 0.08	0.09 ± 0.08	0.35 ± 0.07
B-3	0.07 ± 0.59	0.02 ± 0.01	0.02 ± 0.02	0.08 ± 0.02	0.10 ± 0.01	0.01 ± 0.02	3.11 ± 1.21	0.78 ± 0.33	0.02 ± 0.01	0.23 ± 0.09	4.45 ± 0.88	0.29 ± 0.09	0.04 ± 0.03	8.92 ± 0.38	8.08 ± 0.92	7.31 ± 0.82	1.15 ± 0.76	0.12 ± 0.02	0.42 ± 0.05	0.11 ± 0.14	0.35 ± 0.04
B-4	0.10 ± 0.05	0.02 ± 0.01	0.02 ± 0.01	0.09 ± 0.07	0.10 ± 0.09	0.02 ± 0.02	3.31 ± 0.97	1.01 ± 0.09	0.01 ± 0.01	0.21 ± 0.04	3.78 ± 1.04	0.13 ± 0.08	0.05 ± 0.02	8.76 ± 1.03	8.16 ± 1.02	6.87 ± 1.32	0.84 ± 0.63	0.14 ± 0.05	0.38 ± 0.11	0.10 ± 0.07	0.33 ± 0.12
B-5	0.09 ± 0.03	0.03 ± 0.02	0.02 ± 0.01	0.10 ± 0.02	0.08 ± 0.04	0.02 ± 0.01	3.57 ± 1.13	1.11 ± 0.21	0.02 ± 0.01	0.14 ± 0.09	3.29 ± 0.59	0.18 ± 0.08	0.06 ± 0.03	8.52 ± 0.71	8.85 ± 1.08	6.53 ± 0.83	1.06 ± 0.85	0.16 ± 0.03	0.45 ± 0.21	0.10 ± 0.12	0.30 ± 0.18
B-6	0.11 ± 0.13	0.01 ± 0.02	0.01 ± 0.01	0.10 ± 0.13	0.07 ± 0.09	0.03 ± 0.01	3.03 ± 0.75	0.69 ± 0.18	0.02 ± 0.01	0.17 ± 0.03	3.67 ± 0.49	0.10 ± 0.05	0.09 ± 0.01	9.16 ± 0.22	9.02 ± 1.57	6.30 ± 0.77	0.94 ± 0.49	0.15 ± 0.11	0.43 ± 0.19	0.09 ± 0.08	0.37 ± 0.09
B-7	0.10 ± 0.08	0.03 ± 0.02	0.02 ± 0.01	0.11 ± 0.19	0.11 ± 0.08	0.02 ± 0.01	3.16 ± 0.32	1.77 ± 0.29	0.03 ± 0.02	0.11 ± 0.09	4.13 ± 0.92	—	0.06 ± 0.01	9.29 ± 0.74	9.08 ± 2.31	5.80 ± 1.92	0.89 ± 0.21	0.17 ± 0.12	0.46 ± 0.11	0.11 ± 0.09	0.32 ± 0.19
B-8	0.11 ± 0.07	0.03 ± 0.01	0.02 ± 0.02	0.10 ± 0.03	0.10 ± 0.09	0.02 ± 0.01	2.93 ± 0.87	0.95 ± 0.39	0.02 ± 0.01	0.13 ± 0.08	4.33 ± 1.01	0.12 ± 0.07	0.07 ± 0.02	9.39 ± 0.48	9.18 ± 2.45	5.86 ± 0.98	0.93 ± 1.05	0.16 ± 0.09	0.48 ± 0.33	0.11 ± 0.03	0.37 ± 0.33

H-1	0.11 ± 0.09	0.02 ± 0.01	0.02 ± 0.01	0.07 ± 0.09	0.10 ± 0.03	0.02 ± 0.01	3.04 ± 0.39	1.17 ± 0.47	0.03 ± 0.01	0.14 ± 0.03	2.08 ± 0.31	0.13 ± 0.01	0.06 ± 0.02	10.21 ± 0.72	10.63 ± 1.39	5.91 ± 1.22	2.93 ± 0.37	0.06 ± 0.03	0.57 ± 0.42	0.08 ± 0.07	0.61 ± 0.16
H-2	0.11 ± 0.12	0.02 ± 0.03	0.01 ± 0.01	0.08 ± 0.11	0.09 ± 0.04	0.01 ± 0.01	3.01 ± 0.78	0.92 ± 0.19	0.03 ± 0.02	0.09 ± 0.02	2.11 ± 0.49	0.09 ± 0.03	0.05 ± 0.01	10.22 ± 0.28	10.83 ± 1.29	5.74 ± 0.93	2.79 ± 0.44	0.03 ± 0.08	0.61 ± 0.48	0.07 ± 0.03	0.61 ± 0.08
H-3	0.12 ± 0.10	0.01 ± 0.02	0.02 ± 0.01	0.09 ± 0.06	0.09 ± 0.06	0.01 ± 0.02	2.81 ± 0.39	0.88 ± 0.31	0.02 ± 0.01	0.07 ± 0.09	1.87 ± 0.71	0.02 ± 0.01	0.07 ± 0.03	9.71 ± 0.33	10.43 ± 1.03	5.51 ± 1.42	2.83 ± 0.67	0.05 ± 0.06	0.50 ± 0.39	0.07 ± 0.11	0.59 ± 0.18
H-4	0.10 ± 0.07	0.02 ± 0.01	0.02 ± 0.01	0.10 ± 0.11	0.08 ± 0.03	0.02 ± 0.01	2.59 ± 0.88	1.19 ± 0.33	0.03 ± 0.02	0.11 ± 0.09	1.38 ± 1.04	0.16 ± 0.07	0.06 ± 0.02	9.72 ± 0.21	10.23 ± 1.78	5.54 ± 1.81	2.59 ± 0.34	0.08 ± 0.13	0.53 ± 0.31	0.05 ± 0.03	0.61 ± 0.19
H-5	0.09 ± 0.03	0.03 ± 0.01	0.03 ± 0.02	0.10 ± 0.12	0.08 ± 0.06	0.02 ± 0.03	2.50 ± 0.89	0.91 ± 0.29	0.03 ± 0.01	0.24 ± 0.02	1.69 ± 0.34	0.20 ± 0.07	0.05 ± 0.01	9.38 ± 0.26	9.63 ± 0.77	5.65 ± 0.62	2.28 ± 0.13	0.06 ± 0.11	0.54 ± 0.21	0.06 ± 0.02	0.51 ± 0.11
H-6	0.09 ± 0.06	0.02 ± 0.01	0.02 ± 0.01	0.11 ± 0.02	0.10 ± 0.05	0.03 ± 0.02	2.80 ± 0.57	0.78 ± 0.39	0.01 ± 1.01	0.14 ± 0.08	1.88 ± 0.82	0.09 ± 0.08	0.04 ± 0.03	9.22 ± 0.49	9.07 ± 0.89	5.20 ± 1.71	2.38 ± 0.39	0.06 ± 0.09	0.55 ± 0.12	0.06 ± 0.04	0.61 ± 0.21
H-7	0.10 ± 0.21	0.01 ± 0.01	0.02 ± 0.03	0.09 ± 0.08	0.11 ± 0.03	0.02 ± 0.01	3.34 ± 0.29	0.89 ± 0.18	0.03 ± 0.02	0.30 ± 0.03	1.77 ± 0.82	0.02 ± 0.01	0.06 ± 0.02	10.29 ± 0.83	9.52 ± 0.71	5.08 ± 1.09	2.47 ± 0.33	0.07 ± 0.06	0.43 ± 0.17	0.03 ± 0.02	0.53 ± 0.10
H-8	0.11 ± 0.09	0.02 ± 0.03	0.02 ± 0.01	0.10 ± 0.08	0.10 ± 0.01	0.02 ± 0.01	3.01 ± 0.82	0.68 ± 0.38	0.03 ± 0.01	0.23 ± 0.09	2.21 ± 0.88	0.08 ± 0.03	0.06 ± 0.02	10.83 ± 0.87	10.06 ± 0.93	4.51 ± 1.72	2.70 ± 0.53	0.06 ± 0.04	0.52 ± 0.32	0.04 ± 0.02	0.55 ± 0.14

**Table 6:** The *P* results of LSD of one-way ANOVA.

Constituents		W	T	B	H
Coniferyl ferulate	W	—	0.000	0.000	0.000
	T	—	—	0.000	0.002
	B	—	—	—	0.013
	H	—	—	—	—
Z-ligustilide	W	—	0.000	0.000	0.000
	T	—	—	0.000	0.000
	B	—	—	—	0.000
	H	—	—	—	—
Senkyunolide A	W	—	0.000	0.001	0.000
	T	—	—	0.000	0.000
	B	—	—	—	0.563
	H	—	—	—	—
Na	W	—	0.000	0.000	0.000
	T	—	—	0.031	0.422
	B	—	—	—	0.005
	H	—	—	—	—
Mg	W	—	0.000	0.000	0.031
	T	—	—	0.000	0.000
	B	—	—	—	0.001
	H	—	—	—	—
K	W	—	0.000	0.993	0.001
	T	—	—	0.000	0.000
	B	—	—	—	0.000
	H	—	—	—	—
Ca	W	—	0.000	0.228	0.000
	T	—	—	0.000	0.372
	B	—	—	—	0.000
	H	—	—	—	—
Mn	W	—	0.000	0.000	0.896
	T	—	—	0.434	0.000
	B	—	—	—	0.000
	H	—	—	—	—
Fe	W	—	0.011	0.000	0.001
	T	—	—	0.002	0.269
	B	—	—	—	0.034
	H	—	—	—	—
Cu	W	—	0.000	0.000	0.149
	T	—	—	0.506	0.000
	B	—	—	—	0.000
	H	—	—	—	—
Zn	W	—	0.000	0.002	0.000
	T	—	—	0.000	0.000
	B	—	—	—	0.000
	H	—	—	—	—

## Data Availability

The data used to support the findings of this study are available from the corresponding author upon request.
